# Selection of Surge Protection Module Components for Communication Lines Using a Genetic Algorithm

**DOI:** 10.3390/s22062075

**Published:** 2022-03-08

**Authors:** Dariusz Zieliński, Damian Grzechca

**Affiliations:** 1 Alstom ZWUS Sp. z o.o., 12 Modelarska Street, 40-142 Katowice, Poland; dariusz.zielinski@alstomgroup.com; 2 The Faculty of Automatic Control, Electronics and Computer Science, The Silesian University of Technology, 16 Akademicka Street 16, 44-100 Gliwice, Poland

**Keywords:** surge protection, genetic algorithm, circuit element selection, lighting protection

## Abstract

Among a variety of problems with communication lines, the faults of surge protection modules in railway applications have a significant impact on the transmission line availability, especially if the devices are located in lightning-prone areas or other high-energy exposure areas, such as voltages or current changes. An advanced optimization of the surge protection module is proposed together with its verification, based on simulated waveforms for components and their limitations (e.g., power, peak power, maximum voltages, maximum currents, etc.). It allows for gathering information about the safety margin for each parameter of the components. This can be used to manage the probability of damage to the protection module. The authors have shown the power distribution during exposure that should be considered while developing new devices for railway transportation industry.

## 1. Introduction

Design of the electronic equipment for use in the railway industry, especially near the track, is a very difficult task, because of the wide spectrum of disturbances and obstacles which results in various requirements that must be fulfilled. Some of them are caused by difficult environmental conditions, i.e., wide working temperature range from −50 °C (Siberian regions) to 85 °C, humidity, and even the risk of flooding in the Netherlands. Additionally, a train is powered up by high voltage (up to 25 kV [1,2]) and such a power line generates a huge electric field [3] which causes disturbances in the operation of electronic equipment. Train acceleration (as speed increases) also has an impact on the current observed [4,5,6] in the surrounding rail infrastructure. Obviously, the railway networks spread over a large area are usually well grounded, but they are also highly exposed to lightning issues [7,8,9,10,11]. Such an environmental condition may cause serious damage to the infrastructure and also to the unprotected or weakly protected electronic devices. The railway communication and safety systems are composed of many electronic wayside devices, such as wheel detectors, signals, and point machines. For safety reasons, all the devices must satisfy highly strict standards and they also must be certified. The cost of certification entails both time and money because the time required to access the certified laboratories and the cost of testing play an important role in the final implementation. Hence, the almost-ready-to-market devices should undergo a testing and certification process. The device design process must take into consideration the duration of the certification process, the number of probable iterations (a single approach is the target), and the so-called good engineering practice. Now, it is clear that a design process is not a trivial task and must take time due to the abovementioned facts. Regulations in force on various markets and railway regions enforce further regulations (the local/national law is very often much more restrictive than the international standards), e.g., a device must function properly in the harsh environmental conditions for at least 25 years. A device can be divided into two main parts: the main, or core, part which is responsible for its functionality, e.g., an axle counting, and the surge protection module.

Taking into consideration the high voltage and lightning issues that a device must survive, an appropriate and robust interface needs to be designed. Lightning has the biggest impact on the device from the electrical point of view. Therefore, it is crucial to design a robust interface (power or communication one) [12] in order to protect the core of the device and protect against damage [13]. In this paper, the authors propose the application of a genetic algorithm [14] for selecting values of elements of the interface hardware. To increase the service life, the application of a standard circuit topology for communication lines (controller area network—CAN) has been considered a good engineering practice. A wide, but limited, and available range of items are preselected for the purpose of discourse. The analysis is performed based on the use of a communication cable model that is introduced in [15].

The authors propose a new approach to designing a protection circuit by introducing railway standards to a typical circuit topology and a genetic algorithm for selecting values of elements from the predefined domain. The standard protection circuit topology connected to a wheel detector via a communication cable (the railway cable model is introduced in [15]) is discussed in the paper. The problem coding is presented in detail together with the limited domain of the values of elements. For finding the suboptimal solution, the GA (genetic algorithm) was launched several times in the Matlab environment. Our goal (fitness) function takes into consideration all the crucial parameters, i.e., power dissipation, peak power, maximum voltages, and currents.

## 2. Problem Description

**Figure 1:**
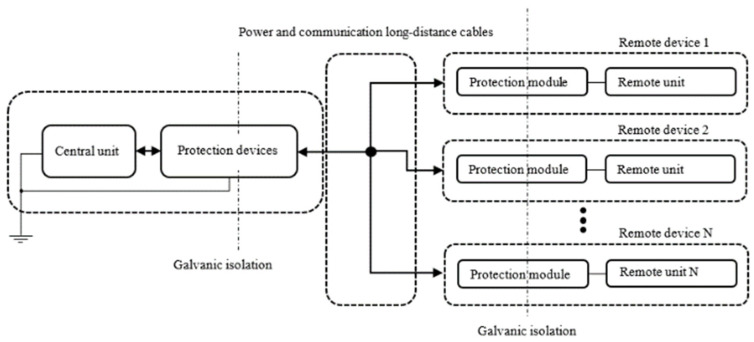
Communication lines topology [15].

Nowadays, serial communication interfaces (e.g., CAN [16]) become widely used in the railway industry, because of the cost efficiency and acceptable transfer rate—a cable of minimum length can be used for connecting of all the devices in bus topology over extensive areas. A general diagram of the wheel detector communication lines topology is shown in Figure 1 [15].

A CPU (i.e., an evaluator) is usually located in a relay room, but wheel detectors (remote units) are mounted on the rails in a distributed manner over long distances. Hence, a new device under development has an interface part based on a CAN driver which is usually in the form of an integrated circuit. Unfortunately, even CAN drivers specific for industrial applications do not fulfill crucial railway requirements and therefore the input circuit needs additional protection which consists of chokes, inductors, resistors, surge arresters, and transils [17]. Moreover, the space for using the protection module is also limited, so there is a need to design it in an optimized way. Besides, the laboratory test only gives the information if the circuit passes or fails a test without information about the degree to which the margin for passing/failing the test has been exceeded. Another problem is that there is no report of what could cause the failure of the protection elements (voltages, currents, or power dissipation). All the mentioned issues are important for an electronic hardware designer, and they should be corrected as soon as possible due to time to market product delivery and the project budget which is always limited. Therefore, it is required and expected that the tests will be passed at the first attempt without a useless iteration scheme.

Due to the abovementioned requirements, there is a need to find out before type tests are started if the designed protection unit will be efficient to pass required tests in compliance with the standards or usually more restricted customer requirements, which could be confirmed by [18]. Authors [19] investigated the impact of lightning and electromagnetic interferences on the trackside devices and proved they had a significant influence on the railway industry. The problem of immunity of railway traffic control equipment is also discussed in [20], where information can be found that an insufficient level of protection can lead to high system maintenance costs.

Mainly, papers discussing lightning immunity analysis of power lines can be found in the literature. In [21], authors presented time characteristics for lightning strike, without analysis of the impact on the powered circuit. 

Paper [22] also presents only a general view of the problem, without any detailed analysis of component parameters and there is no multiple-stage protection; a different group of surge arresters was used because of higher voltages.

## 3. Test Case

One of the valuable test cases to ensure EMC immunity is when a port is exposed to surges. It was selected to find an optimum solution for the presented problem, due to good estimation of exposure in railway applications. 

The test environment is described in [23]. Immunity of I/O ports must be tested with the circuit test voltage 1 kV (1.2 µs/50 µs) for line-to-line configuration (differential mode). It also refers to [24], which gives more precise information and could be used in other sectors of industry. Therefore, generally, this method could be used for other kinds of applications (e.g., automotive, green energy, robots, etc.).

**Figure 2:**
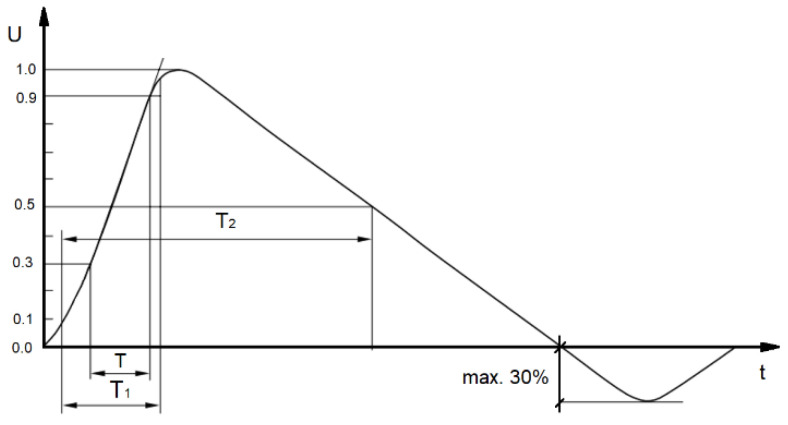
Surge waveform of open-circuit voltage based on [23].

Standardized waveform shape of surge is shown in Figure 2.

There is no precise time of exposure defined in standard [23], but only partial information is presented in Figure 2. There are T1 and T2 defined, when the biggest energy is emitted, without notification about total signal duration. According to that, basic time of exposure was set to 150 µs, based on laboratory experience, but due to the presence of capacitors and inductors (energy storage elements), the total time was extended up to 300 µs to cover possible module reaction. The exposure time was doubled. It is considered as the worst-case scenario because when the duration of exposure is increased, the maximum parameters (voltages, currents, and power dissipation) will decrease, which represents the highest safety margin in a real environment.

Moreover, customers require higher levels of exposure in commercial tenders than defined in the abovementioned standards, due to the awareness of the hazards in railway applications. Therefore, it is desirable to design a dedicated protection module for all the offered devices, taking the project limitations (available space, costs, amplitude of exposure, etc.) into account. Our entry project limitations are exposure to 6 kV surge applied to a differential mode. The railway standard specified in [23] is exceeded by 600%, which simply increases the reliability of the devices.

## 4. Protection Circuit Topology and Its Parameter Domain

**Figure 3:**
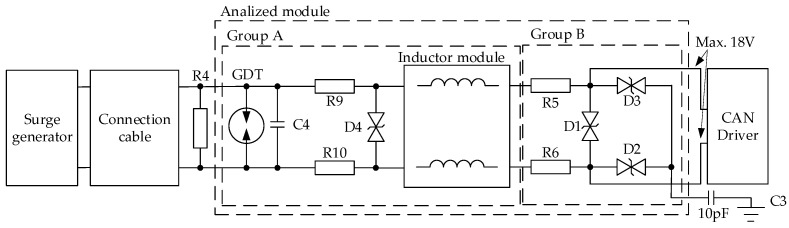
Diagram of the analyzed circuit.

A typical protection circuit scheme is presented in Figure 3. It is excited by the surge generator [25] and a wayside transmission cable model described in [15]. The presented surge generator was verified during preliminary tests. The simulation shape, amplitude, and duration meet the requirements presented in [23]. It must be emphasized that the cable model was designed at the earlier stage of the research. Its length has been set to a segment of 10 m. The length comes from two issues. The first one is the minimum but reliable distance between the device and the point of lightning effect. It is unusual for a device to be hit directly by lightning, and the second one is the proposed model for a railway infrastructure cable. In [15], the minimum segment is set to 10 m. Hence, the authors have decided to combine both of the abovementioned issues and apply such a segment as the worst-case scenario. Additional cable should reduce the protection circuit exposure.

Next, the protection circuit is introduced, and its schematic diagram is divided into two groups due to the fact that the elements from group A might be repaired, whereas elements from group B are hardly accessible. Such an assumption is highly specific, and it is important for the already designed and certified railway equipment. 

Hence, group A is located in the cable connection module and can be replaced. The second group, B, is integrated with a wayside device, and it is irreparable. Thus, it is foreseen and highly probable that the first group of elements might be damaged, contrary to the second group for which the probability should be low due to cost reduction of the maintenance. Such reality enforces additional constraints on parameters from group B. The authors propose to extend the analysis of elements from group B by decreasing the limits for crucial parameters by 50%, i.e., the maximum power dissipation on an element has 50% less than the one declared by the manufacturer.

**Figure 4:**
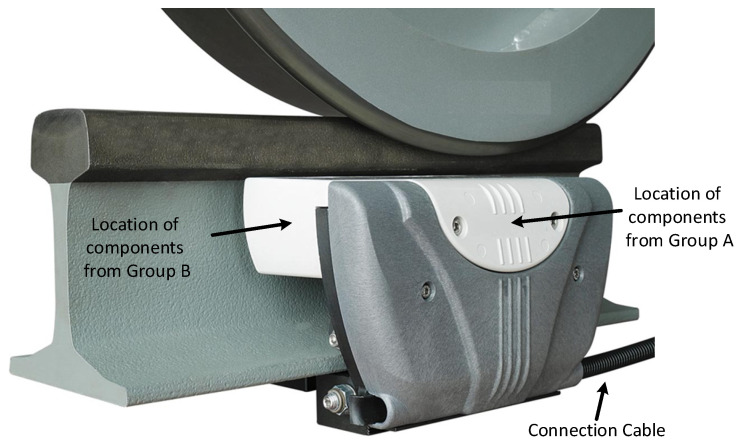
Photo of a wheel detector with groups indicated.

A real configuration of components location, as well as the position of a wheel detector on a rail, is shown in Figure 4.

In group A, all coefficients for the parameters considered will be increased by 10%. Adaptation of the valuation parameter in both groups could be used according to application requirements. Coefficients for group A and group B were selected according to data presented in [26].

The protection circuit is shown in Figure 3, and it contains the following:

- R4 is a CAN terminator of 120 Ω;
- C3 is the capacitance between the wayside device and a ground plate (its value is estimated based on experience, i.e., during similar tests at the laboratory);
- An inductor module is a specially designed circuit for reducing current emission;
- Gas discharge tube (GDT part number SG75 [27]) is a surface-mounted device (cost- and space-saving optimized) with the lowest possible value of threshold, which is 75 V. According to [27], GDT has only current limitations, which was declared by the manufacturer to amount to 2 kA.

For all parts in group A and group B, crucial parameters were defined according to data sheets or general information presented by the manufacturers.

### 4.1. Transils (D1, D2, D3, and D4)

**Figure 5:**
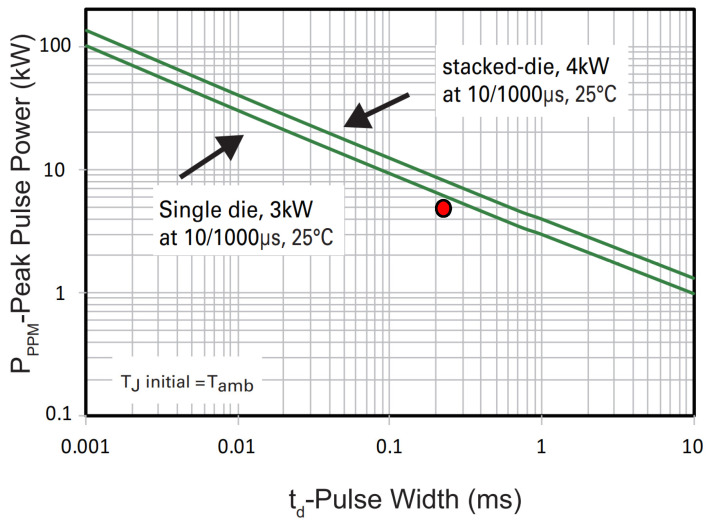
TVS maximum peak pulse power [28].

Based on peak pulse power characteristics presented in a data sheet [28], maximum power dissipated was selected to 5 kW according to Section 3: time of exposure was 300 µs = 0.3 ms (red dot in Figure 5). Bidirectional diodes from page 2 of [28] will be investigated in the research. A total of 32 parts from position SMDJ5.0CA to SMDJ58CA will be taken. Diodes with lowest threshold voltages will be used for direct protection of CAN driver and with the highest threshold value that could be selected for the preliminary stage of protection (D4). Current limitation for transils is provided by the manufacturer in the data sheet [28].

### 4.2. Resistors (R5, R6, R9, and R10)

Considering resistors and the assumed pulse duration of the surge, the maximum total power dissipated by a single resistor can be read from the characteristic depicted in [29]. The red dot indicates the maximum power point for a resistor size 2512 and pulse duration of 300 µs. It is clearly visible that the maximum total power cannot exceed 900 W [29].

Additionally, peak power for a resistor must be below the required level. To calculate this, a plot presented in [29] can be used. For resistance value from 1 Ω to 20 Ω, the characteristic is linear (case 2512), so maximum power peak for exposure 1.2 µs/50 µs using equation 1 could be set to 2500 W. This value must be checked by GA to ensure appropriate working conditions for resistors.

(1) $$P=\frac{U^{2}}{R},$$

- P—power dissipated on considered resistors, i.e., R5, R6, R9, and R10;
- U—voltage on the considered resistor;
- R—resistance of the considered elements.

Due to design role and cost optimization (bill of material standardization), resistors must be the same, e.g., R9 = R10, R5 = R6, and D3 = D2. Maximum sum of resistance (R9 + R10 + R5 + R6) due to company’s internal requirement was set to 18 Ω, because higher value could cause transmission breaks over longest distances. 

The set of available resistors in terms of R9 and R10 is 1 Ω, 1.2 Ω, 1.5 Ω, 1.8 Ω, 2.2 Ω, 2.7 Ω, 3.3 Ω, 3.9 Ω, 4.7 Ω, 5.1 Ω, 5.6 Ω, 6.2 Ω, 6.8 Ω, 7.5 Ω, 8.2 Ω, and 9.1 Ω. The set of resistors in terms of R5 and R6 is limited to the range from 1 Ω to 3.9 Ω. Both ranges come from company internal procedures, which enable to use the communication line over long distances, i.e., up to 4 km. It must be emphasized that the number of elements in both sets is 16 for R9 (and also R10) and 8 for R5 (and R6). The number of elements in the domain as a power of 2 allows to use a binary code of 4 and 3 bits in the chromosome.

### 4.3. Capacitor (C4)

The capacitor has one significant limitation described in the data sheet, i.e., the maximum voltage defined as 150% of its nominal value. The set of available capacitances in terms of C4 was limited to 1.0 nF, 2.2 nF, 3.3 nF, 4.7 nF, 5.6 nF, 6.8 nF, 8.2 nF, and 10 nF. This value was used due to available space and technology limitation. Maximum dimensions of a capacitor are restricted by the size of cable connection unit. Where there are components from group A located, larger capacitors will not fit in the area.

### 4.4. Component Limitations

All the component limitations and available specification, based on the data presented before, are provided in Table 1.

All of the above presented component limitations will be verified in the time domain, after finding a suboptimal solution for the discussed problem.

## 5. Algorithm Description

Selecting appropriate elements of a protection circuit is a time-consuming process. Considering the circuit shown in Figure 3 and a limited number of element values, the total number of 33,554,432 cases must be evaluated. A single circuit analysis takes about 39 s, and therefore the total time required for the entire process equals 41 years and 6 months. It is totally out of the question, so there is a demand to make this process shorter. Authors propose a genetic algorithm for optimization and selection the appropriate values of elements. 

**Figure 6:**
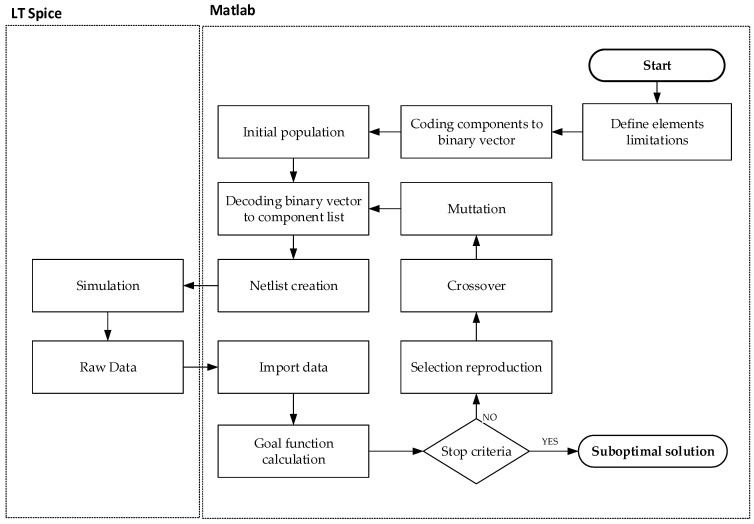
Implemented algorithm flow.

A general diagram of the presented idea is shown in Figure 6. To select parameters for a protection circuit, the Matlab environment and LT Spice simulator were used. An electronic circuit simulator is called from the Matlab script and returns output simulation file which is further processed by Matlab. Based on the obtained data, the fitness function can be calculated to evaluate the population individuals. 

All the data presented in Section 4 are used in algorithm implementation. The main part of the algorithm used to find suboptimal solution is a genetic algorithm with a predefined start option. Population size was set to 50, in line with [32]. Additionally, a high-performance SPICE software was used to simulate a tested circuit. Functional breakdown is presented in Figure 6. 

### 5.1. Binary Input Vector

GA operates on genes represented by binary vectors. Thus, the problem coding is essential and requires a more detailed description. A chromosome presented below is composed of bits which have the following explanation:

- Bits 1:4—representing resistors R9 and R10 simultaneously. Each binary number corresponds to the following resistance in the predefined set (see Section 4.2);
- Bits 5:7—representing resistors R5 and R6 simultaneously. Each binary number corresponds to the following resistance in the predefined set (see Section 4.2);
- Bits 8:12—corresponding D4 to one of 32 diodes (see Section 4.1);
- Bits 13:17—corresponding D1 to one of 32 diodes (see Section 4.1);
- Bits 18:22—corresponding D2 and D3 simultaneously to one of 32 diodes (see Section 4.1);
- Bits 23:25—representing C4 possible configuration described in Section 4.3.

Graphical representation of the input binary vector is presented below:


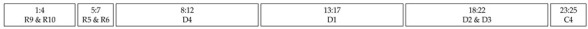


The above described gene codes the protection circuit netlist elements and its models. Therefore, it is possible to create a netlist based on such a binary word and known circuit topology and next perform a simulation in LT Spice. 

Furthermore, the genetic operations coming from a genetic algorithm can be applied. The following settings for GA were utilized:

- Selection specifies how the genetic algorithm selects parents for the next iteration. The default option is set to “selectionstochunif”, i.e., parents are chosen randomly with uniform distribution;
- Reproduction describes how the genetic algorithm creates the next generation. “EliteCount” is corresponding to the number of the best parents, which will be used in the next generation. It was set to 5% of population size;
- Crossover options specify creating crossover child from parents, for the next generation. It was set to “crossoverscattered”, which uses a random binary vector to combine two individuals, to form a child for the next generation;
- Mutation is responsible for chromosome defects; it is set to “mutationgaussian”, which uses Gaussian distribution to make small changes to the parent vector.

### 5.2. Fitness Function Description

The goal of fitness function is to find a suboptimal specification of protection module with preselected components and avoid critical overload parameters (voltages, currents and power defined by manufacturer for a particular component). Therefore, the fitness function evaluates the solution provided by GA and takes the voltages, currents, and power dissipation of an element $\left(M_{k}^{i}\right)$ into account and compares the obtained values to maximum conditions (maximum stress in terms of voltage/current/power ${\hat{M}}_{k}^{i}$). The denominator has a valuation parameter ($\beta_{k}^{i}$) in order to limit any damage of the most critical elements. Parameter α is penalty factor, it is introduced if a single parameter exceeds its maximum value (${\hat{M}}_{k}^{i})$. Working beyond the range of this value ${\hat{M}}_{k}^{i}$ causes damage to the component, and the test fails.

Based on that, a fitness function was defined as Equation (2):

(2) $$fitness=\left(\overset{N_{e}}{\underset{k=1}{\sum }}\overset{N_{p}}{\underset{i=1}{\sum }}\frac{M_{k}^{i}}{\beta_{k}^{i}\cdot {\hat{M}}_{k}^{i}}\cdot \alpha \right),$$

- $M_{k}^{i}$—a calculated value of *i*-th parameter for component *k*, e.g., power peak for R9;
- ${\hat{M}}_{k}^{i}$—a maximum value of *i*-th parameter for component *k*, e.g., voltage, power, or current defined in the manufacturer documentation without margin;
- $\beta_{k}^{i}$—a valuation parameter in respect of components in group A (Figure 3) it can be replaced with the maximum ratio factor (110% of maximum value [26]). In the case of the other one (group B), it was defined as 50% ([26]) of the value declared by the manufacturer. This factor represents the valuation parameter of component damage [26];
- $N_{p}$—number of analyzed parameters per single component, e.g., for R9 $N_{p}$ = 3, namely average power, peak power, and voltage. i = 1, …, $N_{p}$, where i is the following index of the simulated parameter;
- $N_{e}$—number of analyzed components, $N_{e}=9$ in the diagram presented (Figure 3) Indicator k  = 1, 2, …9;
- α—penalty factor can take values 1 or 26, i.e., if $M_{k}^{i}\leq {\hat{M}}_{k}^{i}$ then α=1, otherwise α=26. The penalty factor (α) has been set to 26, so if the value of a parameter is above the limitation multiplied by ratio factor, the goal function will take 26 instead of the ratio of the calculated value to the limit to avoid a situation when one component is overloaded but there are no limitations for the others. Calculation is presented in Table 2.

Hence, the global solution to the problem was defined to minimize the fitness function. Acceptable solutions are within 0 and 26 because

- fitness →0—stands for a circuit with infinitely large limitation, i.e., the result equaling zero is impossible;
- fitness →26—stands for a circuit with all the components working with maximum limitation;
- fitness>26 indicates an unsuitable solution due to fact that a single parameter exceeds its critical margin.

**Table 2:** Calculation of penalty factor (α).

Group	Component	Factory Ratio	Voltage Limitation	Current Limitation	Average Power Limitation	Peak Power Limitation	Number of Critical Parameters
A	C4	110%	✓				1
	GDT	110%		✓			1
	R9	110%	✓		✓	✓	3
	R10	110%	✓		✓	✓	3
	D4	110%		✓		✓	2
B	R5	50%	✓		✓	✓	3
	R6	50%	✓		✓	✓	3
	D1	50%	✓	✓		✓	3
	D2	50%	✓	✓		✓	3
	D3	50%	✓	✓		✓	3
Maximum sum of resistors in lines (R9 + R10 + R5 + R6)							1
					**Total Sum:**		**26**

Stopping criteria for genetic algorithm are presented in Table 3, they are used to stop the execution of algorithm, if some of the conditions occur.

Max stall generations are the main stopping criteria parameters, which are connected with function tolerance. It stops the execution of script when there is no progress in finding a better solution. Other criteria are configured to avoid situation of infinite loop.

## 6. Result Discussion

During the research, many iterations were performed, during the implementation of the algorithm and the verification of its individual versions. Considering that the selection of components is critical for the operation of the device and undoubtedly due to high cost of certification process, the presented solution should be treated as preliminary. It must be verified in a certificated laboratory.

**Figure 7:**
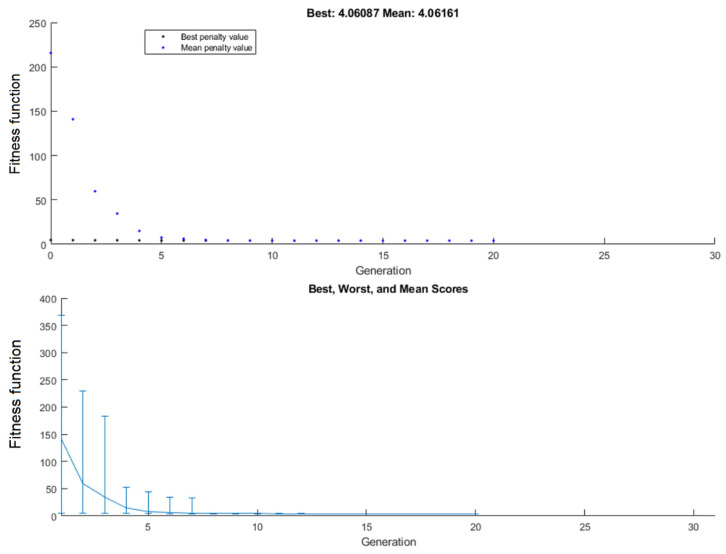
Best, worst, and mean genotypes during optimization process. General historical statistics of genetic algorithm.

All the tasks were performed on a PC computer: AMD Ryzen 5 3600 MHz, 16 GB DDR4. To speed up the system (read/write operations), the disk CORSAIR Force MP600 was used. It increased the speed of algorithm run by ca. 10%. A single iteration containing 50 individuals (1 population) is completed in ca. 32 min (floating). The processing of 20 populations consisting of 1051 individuals takes about 10 h 51 min. The suboptimal solution is presented below. Algorithm finished after 1051 iterations, because of the average change in the fitness function value lower than options: function tolerance. The proposal for the implementation of the following components is presented in Table 4. Statistic data (best, worst, and mean penalty value) for iterations of the genetic algorithm are shown in Figure 7. Based on these, the algorithm from the random set of input vectors selects the best individuals and uses them for creating the new, better collection (mean scores of the fitness function are descending).

**Figure 8:**
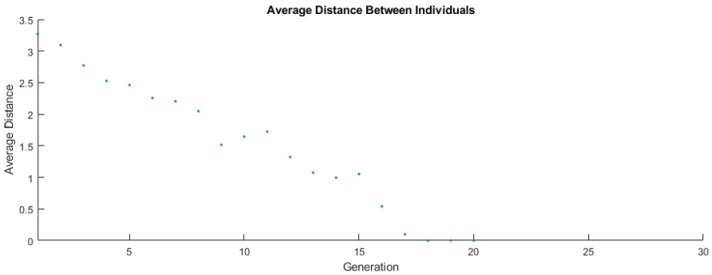
Average distance between individuals for each generation.

In the first few generations it was observed that the maximum operating parameters (value of fitness function above 26) were exceeded in respect of the components. Generally, the course of the function is in line with the authors’ expectations, because the time needed to determine a solution close to suboptimal (11 iterations = average distance between individuals over 1.5) is comparable to the time of its verification (9 iterations = average distance between individuals less than 1.5). These statistics of input vectors are shown in Figure 8.

Average distances between individuals were decreasing during the algorithm flow, which confirmed the proper design of the target function for achieving the best suboptimal, which is presented in Table 4. For some generations (e.g., 11th and 15th), the distance between individuals was bigger than previously. This probably results from the randomized part of the genetic algorithm, and it meets the authors’ expectations. In the case of the last three generations (Figure 8), it could be observed that the algorithm worked on very similar input vectors, which was the best result of previous iterations of GA. Data shown in Figure 7 and Figure 8 are convergent, because small changes in input vectors do not increase the fitness function significantly.

Calculated value of the fitness function for the specification (Table 4) is 4.061. It is within the expectation range described in Section 4.4 (value between 0 and 26). All the components did not overload the margin (percentage value below 100%). Moreover, in the case of the more restricted group B (R5, R6, D1, D2, and D3), where the safety margin was set to 50%, there was no danger of failing a test. A total of 78.63% for maximum voltage of D1 means that this parameter reached only 39.315% of the value declared by the manufacturer. High value of power peak for C4 confirms proper application of the component, its main task being to reduce the quickly rising voltages. Therefore, the role of the capacitor will probably be more important during the exposure to burst, when very fast signals (50 ns) are involved in the testing process. The total power dissipation in group A (without GDT) is 12.45 W, and in group B is 5.72 W. It means that in the first group, a higher probability of element’s fault can be expected, and it is in line with the assumptions. Furthermore, the total resistance in the protection module (5.6 Ω) is below the required value, 18 Ω.

The suboptimal solution found was investigated by means of verification of time signal parameters of components, as presented below.

**Figure 9:**
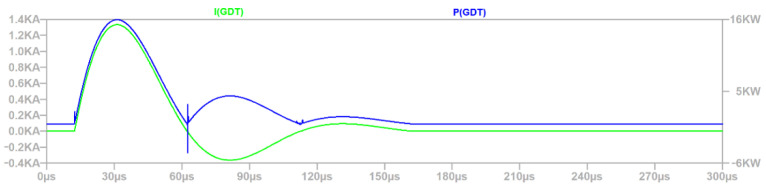
Power and current waveforms of GDT.

Based on time signals shown in Figure 9 a major part of the surge energy is dissipated on GDT. What does this suggest about geometrical position of elements? They should be as close as possible to cable input, because it minimizes the influence of electromagnetic field generated by the large current flow. Maximum current value (1.33 kA) for GDT is below the maximum value declared by the manufacturer (2 kA).

**Figure 10:**
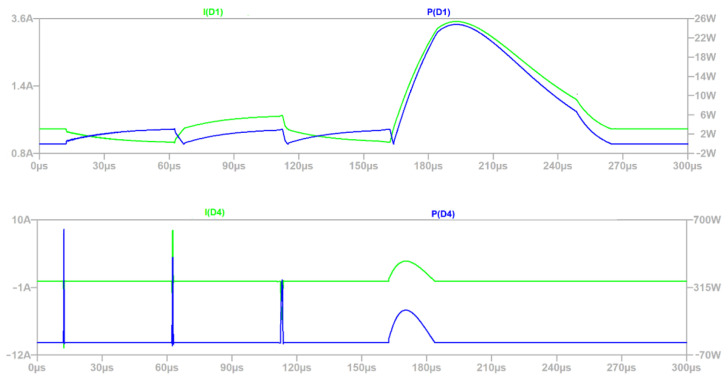
Power and current waveforms of D1 and D4.

Power dissipation for transils D1 and D4 is shown in Figure 10. The peak current observed for D4: SMDJ48CA is 10.868 A against 194.0 A declared by the manufacturer. It follows from the simulation that

- D1: SMDJ6.0CA peak current is 3.505 A vs. 1456.5 A (limit value declared by the manufacturer);
- Peak power for D4 is 646.14 W (maximum 5 kW), and for D1 is 24.80 W (maximum 5 kW).

Based on the above, power and current limitations provided by the manufacturer for respective elements have been met. The waveforms are consistent with the assumption that the load of D1 is smaller than D4. Peak current arrested by D4 is observed because of delay in GDT’s response, and power dissipation of D1 is present because at this time D4 and GDT are not active—voltages in the module are below the threshold of GDT and D4.

**Figure 11:**
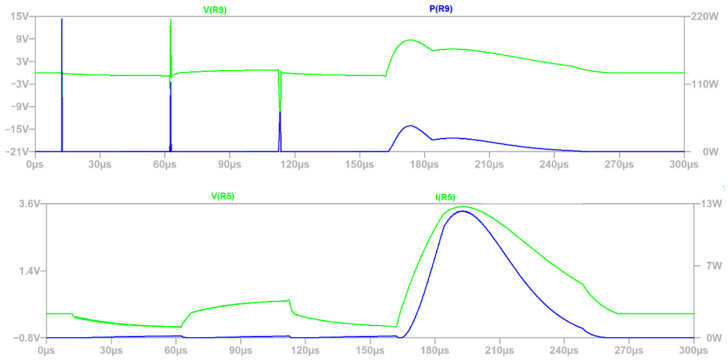
Power and voltages waveforms of R9 and R5.

The instantaneous power plot (Figure 11 of R9 and R5 corresponds to transil currents flows (D1 and D4). It results from the simulation that the maximum voltage for R9 is 19.72 V, which is below the margin declared by the manufacturer (500 V); moreover, the peak power 216.03 W is below 2.5 kW. Again, in this case, the manufacturers’ limitations have been met (Table 1).

**Figure 12:**
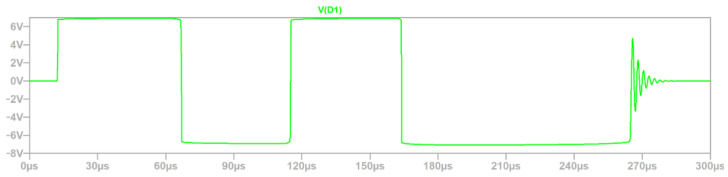
Voltage waveforms of D1.

The voltage on D1 (Figure 12) transil corresponds to the voltage between CAN_H and CAN_L (Figure 3) signals of CAN driver, which is the most sensitive part of the analyzed circuit, but it could be observed that the value is below the margin 9 V = 18 V × 0.5 (Table 1 and protection coefficient factor for group B). The voltage between the inputs of CAN drivers reaches only 7.08 V in the presented simulation.

Based on the waveforms from Figure 9, Figure 10, Figure 11 and Figure 12, the simulation time 300 µs was selected properly, because the defined surge excitation disappeared by this time.

Moreover, the configuration of GA parameters described in Section 5 was verified. The main factors are presented in Table 5.

Increase of the size of the population to 200 significantly extends the time of the processing algorithm, with a slight improvement of fitness function (−0.15%). Setting the size of the population to 120 does not give the expected result, a change in fitness function +2.59%. Decrease in the size of the population size (configuration 2) slightly reduces data processing time but the impact on the fitness function is quite big (+3.25%). Results for the population size 50 and 80 are similar, so due to optimization of algorithm development (shorter time of processing one population), 50 was selected. It needs to be mentioned that GA is a nondeterministic algorithm; it can give varying results and consumes different amounts of time.

The presented component specification (Table 4) is within the safe margin for voltages, currents, power distribution, etc. Despite the increased exposure (6 kV), the protection module enables to disperse the surge disturbance without any damage of elements. Moreover, 6 kV is much higher than the exposure presented in the standard, but many railway markets require values beyond the ones stipulated in the relevant standard. A CAN driver is the most critical part of the unit, and owing to appropriate fitness function design, it is well protected. Based on the simulation, it can be stated that the voltage on D1 reaches only 7.08 V, i.e., 39.32% of the maximum value, which is allowed by the manufacturer for this integrated circuit. Thus, the peak power for D4 (646.14 W) is much higher than for D1 (24.80 W) and it is in line with the expectations of the authors. Components from group A can be easily replaced at a lower cost.

## 7. Conclusions

The article presents a new approach to selection of components of protection modules used for increasing the EMC immunity of the devices which can be used in various sectors of industry, not only in the railway industry, but which can be deployed to a great degree in this environment in particular. The algorithm makes finding the solution possible within a satisfactory period of time, which is less than 11 h. This enables its use in real ongoing and future projects and brings tangible benefits, e.g., minimizes the number of iterations in EMC tests and reduces time spent launching the product, thereby lowering the cost of the R&D process. 

The solution presented is very flexible and can be adapted to meet new requirements, e.g., in terms of temperature, size, and cost constraints. Moreover, it can be more effective in complicated protection modules, where the total number of test cases might reach billions. This algorithm may also reduce the time needed to be ready to offer devices conforming to all the required standards and reduce the volume of R&D expenditures involved. It is planned to use this method in the new projects to be delivered by Alstom because of the advantages described above and the increased awareness of the level of safety margins of the elements. The verification of the proposed specification is planned at the laboratory in a representative test case.

## Figures and Tables

**Table 1:** Component limitations.

Component	Voltage [V]	Current [A]	Power Dissipation [W]	Peak Power [kW]	Possible Configuration
C4	3 kV × 1.5 [30]	Lack of data	Lack of data	Lack of data	Description in Section 4.3
GDT	Lack of data	2 k [27]	Lack of data	Lack of data	SG75
R9	500 [29]	Related to peak power [Section 4.2]	900	2.5	Description in Section 4.2.
R10	500 [29]	Related to peak power [Section 4.2]	900	2.5	Description in Section 4.2.
D4	Related to power	Depends on the type [28]	Lack of data	5.0 (Figure 5)	Description in Section 4.1.
R5	500 [29]	Related to peak power [Section 4.2]	900 W	2.5	Description in Section 4.2
R6	500 [29]	Related to peak power [Section 4.2]	900 W	2.5	Description in Section 4.2.
D1	18 [31]	Depends on the type [28]	Lack of data	5.0 (Figure 5)	Description in Section 4.1.
D2	18 [31]	Depends on type [28]	Lack of data	5.0 (Figure 5)	Description in Section 4.1.
D3	18 [31]	Depends on the type [28]	Lack of data	5.0 (Figure 5)	Description in Section 4.1

**Table 3:** Stopping criteria order.

Option	Value	Description
Max Generations	30	Defines maximum number of generated population during algorithm execution.
Max Time	24 × 3600 [s]	Maximum amount of time (in seconds) consumed by Matlab for internal data process (import data, goal function calculation, GA operation, create netlist for next simulation).
Function Tolerance	0.001	Difference between goal function calculation for following members of generations, below this factor the number of stall generations goes up. Value is set to 0.1%.
Max Stall Generations	10	Minimum number of stall generations which indicates stop to find a better solution. In this case 10 following goal function calculations with differences less than 0.1% (Function Tolerance = 0.001) will stop the execution of algorithm.

**Table 4:** Proposal of suboptimal components specification.

Component Designator	Proposed Value	Peak Voltage [V]	Peak Current [A]	Power Dissipation [W]	Power Peak [W]	Percent. of Max. Voltage	Percent of Max. Current	Percent. of Max. Diss. Power	Percent of Peak Power
C4	2.2n	2083.09	10.73	2.27	5726.72	42.08%	0.00%	0.00%	0.00%
GDT	SG75	98.75	1335.72	1303.29	15,974.54	0.00%	60.71%	0.00%	0.00%
R9	1.8R	19.72	10.96	3.11	216.03	1.99%	0.00%	0.31%	7.86%
R10	1.8R	19.58	10.88	3.11	216.03	1.98%	0.00%	0.31%	7.86%
D4	SMDJ48CA	59.45	10.87	6.23	646.14	0.00%	5.09%	0.00%	11.75%
R5	1R	3.51	3.51	1.11	12.32	0.78%	0.00%	0.25%	0.99%
R6	1R	3.51	3.51	1.11	12.32	0.78%	0.00%	0.25%	0.99%
D1	SMDJ6.0CA	7.08	3.51	3.49	24.80	78.63%	0.48%	0.00%	0.99%
D2	SMDJ6.0CA	6.78	0.06	0.01	0.38	75.32%	0.01%	0.00%	0.02%
D3	SMDJ6.0CA	6.80	0.08	0.01	0.56	75.60%	0.01%	0.00%	0.02%
	Total resistance	5.6 Ω							

**Table 5:** GA parameters and result comparison.

Configuration	Population Size	Function Tolerance	Time of Processing	Fitness Function	Percentage Change of Fitness Function Related to Conf. 1
1	50	0.001	10 h 51 min	4.061	0.00%
2	20	0.001	8 h 51 min	4.193	+3.25%
3	80	0.001	24 h 34 min	4.080	+0.47%
4	120	0.001	26 h 10 min	4.166	+2.59%
5	200	0.001	25 h 50 min	4.055	−0.15%
6	50	0.01	9 h 40 min	4.097	+0.89%
7	50	0.0001	24 h 10 min	3.982	−1.95%

## Data Availability

The data presented in this study are available on request from the corresponding author. The data are not publicly available as a nondisclosure agreement is required.
